# Dr. Prince A. Morrow

**Published:** 1913-05

**Authors:** 


Dr. Prince A. Morrow, N. Y. University Medical College, 1874, died suddenly in New York, March 17, aged 66. He was well known as a dermatologist and for his work in genito-urinary diseases, being of a generation when these branches had not been separated so sharply as at present. His writings on the social aspects of venereal diseases were also valuable. Some years ago he made a special study of leprosy at Molokai, involving a degree of heroism none the less creditable because no unfortunate result occurred.

